# High-quality nuclear genome for *Sarcoptes scabiei*—A critical resource for a neglected parasite

**DOI:** 10.1371/journal.pntd.0008720

**Published:** 2020-10-01

**Authors:** Pasi K. Korhonen, Robin B. Gasser, Guangxu Ma, Tao Wang, Andreas J. Stroehlein, Neil D. Young, Ching-Seng Ang, Deepani D. Fernando, Hieng C. Lu, Sara Taylor, Simone L. Reynolds, Ehtesham Mofiz, Shivashankar H. Najaraj, Harsha Gowda, Anil Madugundu, Santosh Renuse, Deborah Holt, Akhilesh Pandey, Anthony T. Papenfuss, Katja Fischer

**Affiliations:** 1 Faculty of Veterinary and Agricultural Sciences, The University of Melbourne, Parkville, Victoria, Australia; 2 Bio21 Mass Spectrometry and Proteomics Facility, The University of Melbourne, Melbourne, Victoria, Australia; 3 Cell and Molecular Biology Department, Infectious Diseases Program, QIMR Berghofer Medical Research Institute, Brisbane, Queensland, Australia; 4 Bioinformatics Division, Walter and Eliza Hall Institute of Medical Research, Melbourne, Victoria, Australia; 5 Faculty of Health, School—Biomedical Sciences, Queensland University of Technology, Brisbane, Queensland, Australia; 6 Institute of Bioinformatics, Bangalore, India; 7 Center for Individualized Medicine and Department of Laboratory Medicine and Pathology, Mayo Clinic, Rochester, MN, United States of America; 8 Manipal Academy of Higher Education (MAHE), Manipal, Karnataka, India; 9 Menzies School of Health Research, Charles Darwin University, Darwin, Australia; 10 College of Health and Human Sciences, Charles Darwin University, Darwin, Australia; Federal University of Ceará, Fortaleza, Brazil, BRAZIL

## Abstract

The parasitic mite *Sarcoptes scabiei* is an economically highly significant parasite of the skin of humans and animals worldwide. In humans, this mite causes a neglected tropical disease (NTD), called scabies. This disease results in major morbidity, disability, stigma and poverty globally and is often associated with secondary bacterial infections. Currently, anti-scabies treatments are not sufficiently effective, resistance to them is emerging and no vaccine is available. Here, we report the first high-quality genome and transcriptomic data for *S*. *scabiei*. The genome is 56.6 Mb in size, has a a repeat content of 10.6% and codes for 9,174 proteins. We explored key molecules involved in development, reproduction, host-parasite interactions, immunity and disease. The enhanced ‘omic data sets for *S*. *scabiei* represent comprehensive and critical resources for genetic, functional genomic, metabolomic, phylogenetic, ecological and/or epidemiological investigations, and will underpin the design and development of new treatments, vaccines and/or diagnostic tests.

## Author summary

Scabies is a highly significant parasitic disease caused by the mite *S*. *scabiei*. This NTD has a major adverse impact in disadvantaged communities around the world, particularly when associated with secondary bacterial infections and clinical complications. Here we report the first high-quality genome and transcriptomic data for *S*. *scabiei* and explore molecular aspects of *S*. *scabiei*/scabies. This genome (56.6 Mb, encoding ~ 9,200 proteins) provides a solid foundation for fundamental investigations of the molecular biology of the mite, host-parasite interactions and disease processes as well as for translational research to develop new treatments, vaccines and diagnostic tests.

## Introduction

*Sarcoptes scabiei* is a parasitic mite of the skin that causes scabies, one of the commonest dermatological diseases worldwide that results in major morbidity, disability, stigma and poverty [1, 2]. Of the 15 most burdensome dermatologic conditions, assessed in disability-adjusted life years (DALYs), scabies ranks higher than keratinocyte carcinoma and melanoma [3]. The prevalence of scabies can be very high (35%) in disadvantaged communities, including those in remote tropical regions in northern Australia [2, 4]. Scabies is often associated with secondary, opportunistic bacterial infections, a major concern in children in hyperendemic situations [2, 5]. Here, scabies poses a high risk of potentially life-threatening *Staphylococcus aureus* bacteraemia and severe post-streptococcal sequelae [6, 7], including rheumatic fever, heart disease and/or glomerulonephritis, representing a substantial mortality burden [8]. In spite of this knowledge, current epidemiological data underrepresent the actual scabies burden [9] due to an absence of accurate diagnostic tools and serious gaps in disease surveillance. In 2017, WHO’s recommendation to include scabies in the highest NTD category came with an urgent call for research and drug development [10].

There is no vaccine, and only a small number of treatments are used to combat this highly contagious disease. Topical permethrin and systemic/topical ivermectin are ‘broad-spectrum’ compounds of choice [11]. However, permethrin is not recommended for use in infants, and ivermectin is contra-indicated in patients with severely impaired liver or kidney function and the safety of its use in pregnant women and in children of < 15 kg body weight is only beginning to be investigated [12, 13]. Some other agents, such as sulphur, crotamiton, malathion and benzyl benzoate are presently available for topical application in children, but their clinical efficacies and tolerability have not been adequately assessed. Moreover, currently available drugs kill motile stages (larvae, nymphs and adults) of *S*. *scabiei* by interfering with the mite’s muscle function and/or nervous system [14–17]. These drugs often fail because the eggs of the mite are not susceptible to treatment, and drugs have short half-lives in the skin. Thus, eggs can hatch and perpetuate infection. Resistances to drugs are emerging in *S*. *scabiei* [18], which emphasises the urgency of finding novel scabicides to improve the treatment and management of scabies at the individual-patient, household and community levels. The discovery of new scabicides has been challenging, predominantly because of difficulties in producing adequate amounts of the mite for experimentation and drug screening/testing, and also due to a limited understanding of the mite’s biology and how it interacts with its host at the molecular level.

Given these abovementioned challenges, there is an urgent need to search for new drug targets encoded as proteins in the *S*. *scabiei* genome. Although three draft genomes have been assembled and/or annotated for *S*. *scabiei* from different host animals including human, dog and pig [19, 20], all of them are fragmented, limiting their utility for critical fundamental and applied investigations. Here, we report the first high-quality draft genome for *S*. *scabiei*, complemented by its transcriptome, to underpin fundamental and applied investigations of this parasitic mite at the molecular level. This genome is expected to provide a substantially enhanced resource to the research community for genetic, functional genomic, evolutionary, biological, ecological and epidemiological investigations, and a basis for the discovery of new drug and vaccine targets against scabies.

## Results and discussion

### Genome assembly

We sequenced the genome of *S*. *scabiei* var. *suis* from Australia at 114-fold long read and 443-fold short read coverage (S1 Table), producing a final draft assembly of 56.6 Mb (scaffold N50: 2.97 Mb; Table 1) with a mean GC-content of 33.3%. The present assembly was represented by a total of 66 contiguous sequences, compared with 4,268, 3,138 and 18,860 contigs for previous assemblies for *S*. *scabiei* var. *suis*, var. *hominis* and var. *canis*, respectively [19, 20]. As *S*. *scabiei* var. *suis* cells appear to contain 17–18 chromosomes [21], this assembly of 21 contigs (Table 1; L90 = 21 for *S*. *scabiei var*. *suis*) indicates that we have achieved a near chromosomal-level assembly. The estimated repeat content for this genome is 10.6%, equating to 6.0 Mb of DNA. The assembly contained 3.1% (1.8 Mb) interspersed and 7.9% (4.4 Mb) simple and low complexity repeats (S1 Table), the latter of which is in accord with findings for the house dust mite, *Dermatophagoides pteronyssinus* (9.2%; ~ 4.8 Mb) [22]. DNA transposons are more abundant (0.89%; 506 kb) in identified retrotransposon sequences (S1 Table) than long terminal repeats (LTRs) (0.38%; 215 kb), long interspersed elements (LINEs) (0.11%; 61 kb) and short interspersed elements (SINEs) (0.04%; 22kb). We also identified 915 kb (1.7%) of unclassified repeat elements (S1 Table).

**Table 1:** Features of *Sarcoptes scabiei* draft genome.

Description	*Sarcoptes scabiei* var. *suis*	*Dermatophagoides pteronyssinus*	*Tetranychus urticae*	*Psoroptes ovis*	*Sarcoptes scabiei* var. *canis*
NCBI accession identifier	WVUK01000000	GCF_001901225.1	GCF_000239435.1	GCA_002943765.1	GCA_000828355.1
Genome size (bp)	56,576,587	70,778,228	90,828,597	63,414,655	56,262,437
Number of scaffolds	66	1,373	641	134	18,860
N50 (bp); L50	2,965,819; 5	450,436; 33	2,993,488; 10	2,279,290; 8	11,557; 972
N90 (bp); L90	703,488; 21	51,383; 206	732,742; 34	560,979; 29	1,270; 7,002
Genome GC content (%)	33.3	30.9	32.3	28.3	33.3
Repetitive sequences (%)	10.6				
Exonic proportion; incl. introns (%)	28.0; 44.4	26.0; 45.0	19.3; 47.5	23.5; 28.5	21.3; 27.1
Number of putative protein-coding genes	9,174	11,159	11,428	12,037	10,460
Mean; median gene size (bp)	2,735; 1,601	2,852; 1,576	3,836; 1,656	1,501; 1,107	1,459; 1,025
Mean; median CDS length (bp)	1,729; 1,305	1,646; 1,251	1,547; 1,209	1,236; 915	1,146; 830
Mean exon number per protein-coding gene	4.0	3.6	3.9	3.3	3.1
Mean; median exon length (bp)	431; 241	458; 253	396; 196	373; 186	372; 207
Mean; median intron length (bp)	334; 71	464; 71	788; 98	120; 70	147; 71
Coding GC content (%)	37.2	33.1	37.7	33.5	37.6
Number or transfer RNAs	294				
BUSCO completeness: complete; partial (%)	90.8; 92.6	92.3; 93.7	91.5; 92.8	84.5; 87.4	80.8; 87.5

### Gene set

**Fig 1:**
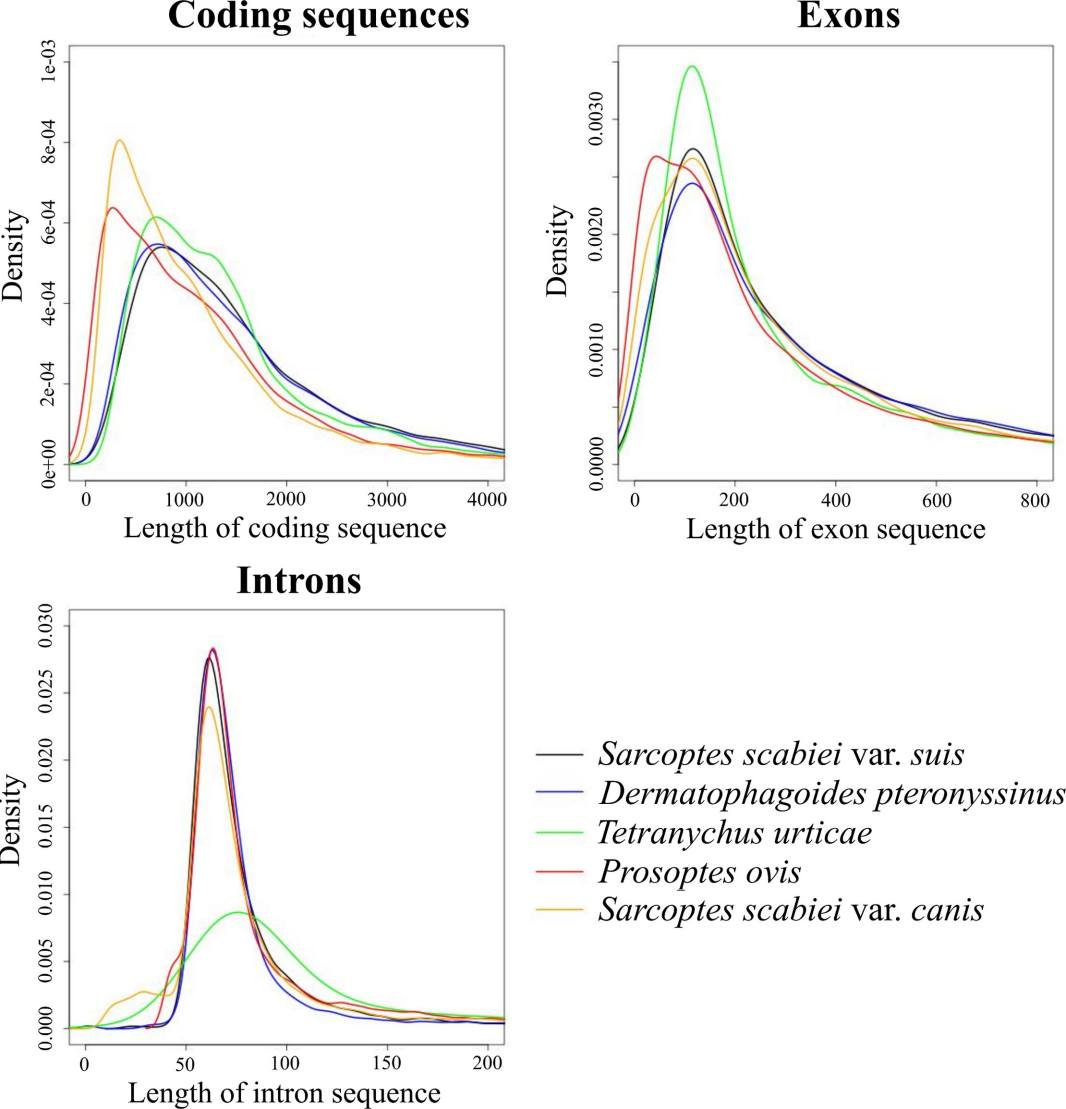
Characteristics of coding sequences, exons and introns. Density diagrams–showing the distribution of data–were used to compare coding sequences, exons and introns for the gene models of the mite species *Sarcoptes scabiei* var. *suis* (black), *Dermatophagoides pteronyssinus* (blue), *Psoroptes ovis* (red), *Tetranychus urticae* (green) and *Sarcoptes scabiei* var. *canis* (yellow). The NCBI accession identifiers for the genomes of the taxa included here are: WVUK01000000, GCF_001901225.1, GCA_002943765.1, GCF_000239435.1 and GCA_000828355.1, respectively.

**Fig 2:**
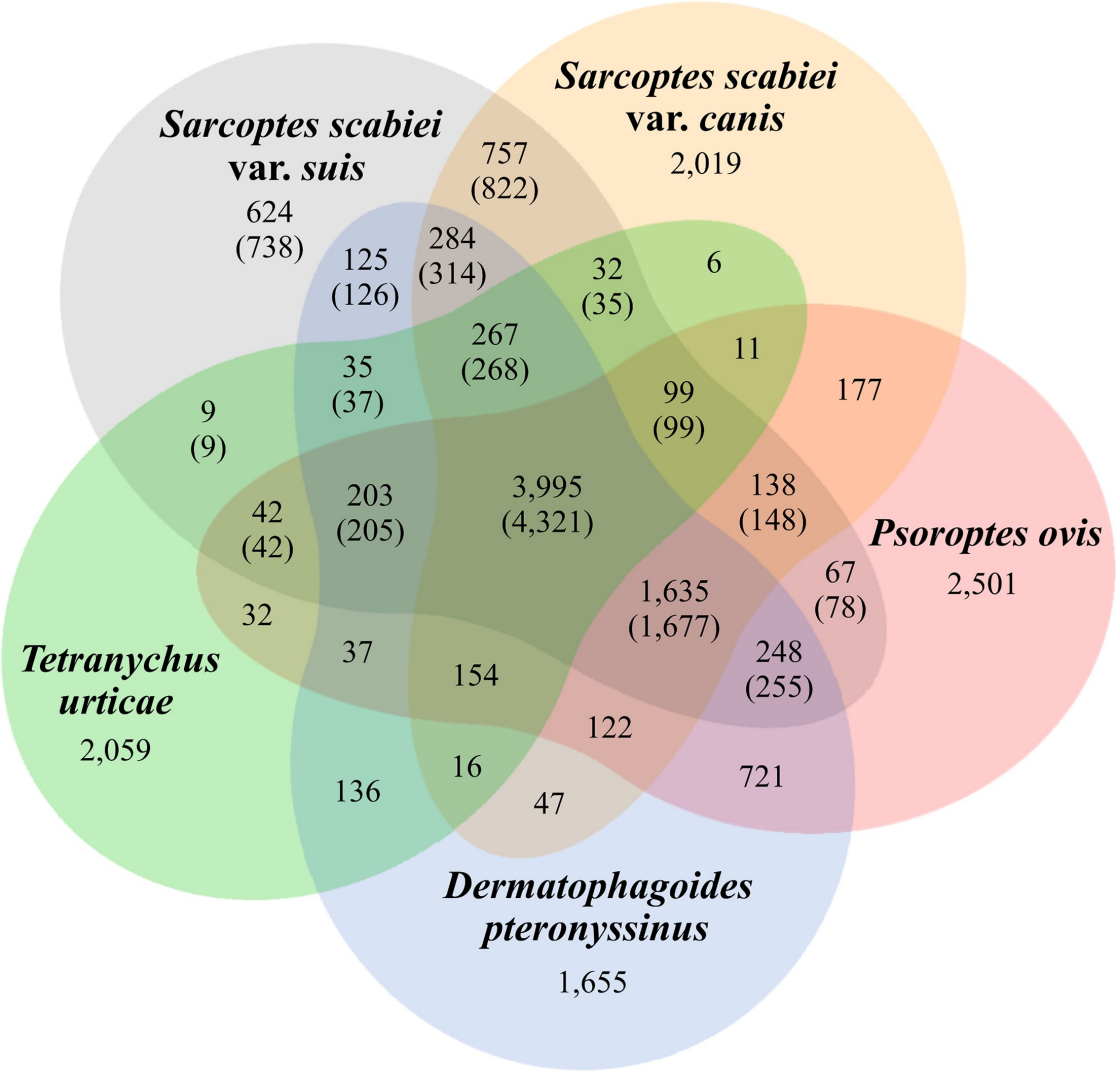
Comparison of orthologous proteins among selected mite species. VENN diagram showing numbers of homologous groups of proteins among *Sarcoptes scabiei* var. *suis*, *Sarcoptes scabiei* var. *canis*, *Psoroptes ovis*, *Dermatophagoides pteronyssinus* and *Tetranychus urticae*. Protein-coding genes of *S*. *scabiei* var. *suis* are indicated in parentheses. NCBI accession identifiers for the genomes of the taxa included here are: WVUK01000000, GCA_000828355.1, GCA_002943765.1, GCF_001901225.1 and GCF_000239435.1, respectively.

Given the fragmentation in published draft genome assemblies of *S*. *scabiei* variants [19, 20], we elected to predict genes and annotate them independently. We used transcriptomic data for egg, and adult stages of *S*. *scabiei* var. *suis* and protein sequences in UniProtKB/SwissProt (14 May 2019) [23] to support gene predictions. In total, we annotated 9,174 protein-encoding genes consisting of ~ 4.0 exons per gene (Table 1; S2 Table). In the predicted gene set, we inferred 967 (90.8%) of 1,066 complete core essential genes using the program Benchmarking Universal Single-Copy Orthologs (BUSCO) [24] for arthropods, which suggested that the genome is near complete. These findings accord with the numbers of BUSCO orthologs for *D*. *pteronyssinus* (984; 92.3%) [25] and *Tetranychus urticae* (975; 91.5%) [26] (Table 1). The statistics for the gene models of *S*. *scabiei* were similar to those of the well-assembled and annotated genome for *D*. *pteronyssinus* [25]: mean/median lengths of gene regions (2,735/1,601 bp), coding sequences (1,729/1,305 bp), exons (431/241 bp) and introns (334/71 bp)–excluding untranslated regions (UTRs)–were comparable with those of *D*. *pteronyssinus* (i.e. 2,852/1,576 bp, 1,646/1,251 bp, 458/253 bp and 464/71 bp, respectively), but distinct from those of *T*. *urticae* in which genes were larger (3,836/1,656 pb) due to longer intron sizes (788/98 bp) and coding sequences (1,547/1,209 bp), but exons (396/196 bp) were shorter (Table 1; Fig 1). Among these three mite species, *S*. *scabiei* shared more orthologous genes (OrthoMCL; BLASTp E-value of ≤ 10^−8^) with the genome of *D*. *pteronyssinus* (n = 7,203; 75.3%) than with that of *T*. *urticae* (n = 4,797; 52.0%) (Fig 2). Conspicuous are 822 protein-encoding genes (9.6%) that are unique to *S*. *scabiei* (Fig 2) for the acarines compared; 47 of these genes encode excretory/secretory (ES) proteins.

### Genetic relationships

**Fig 3:**
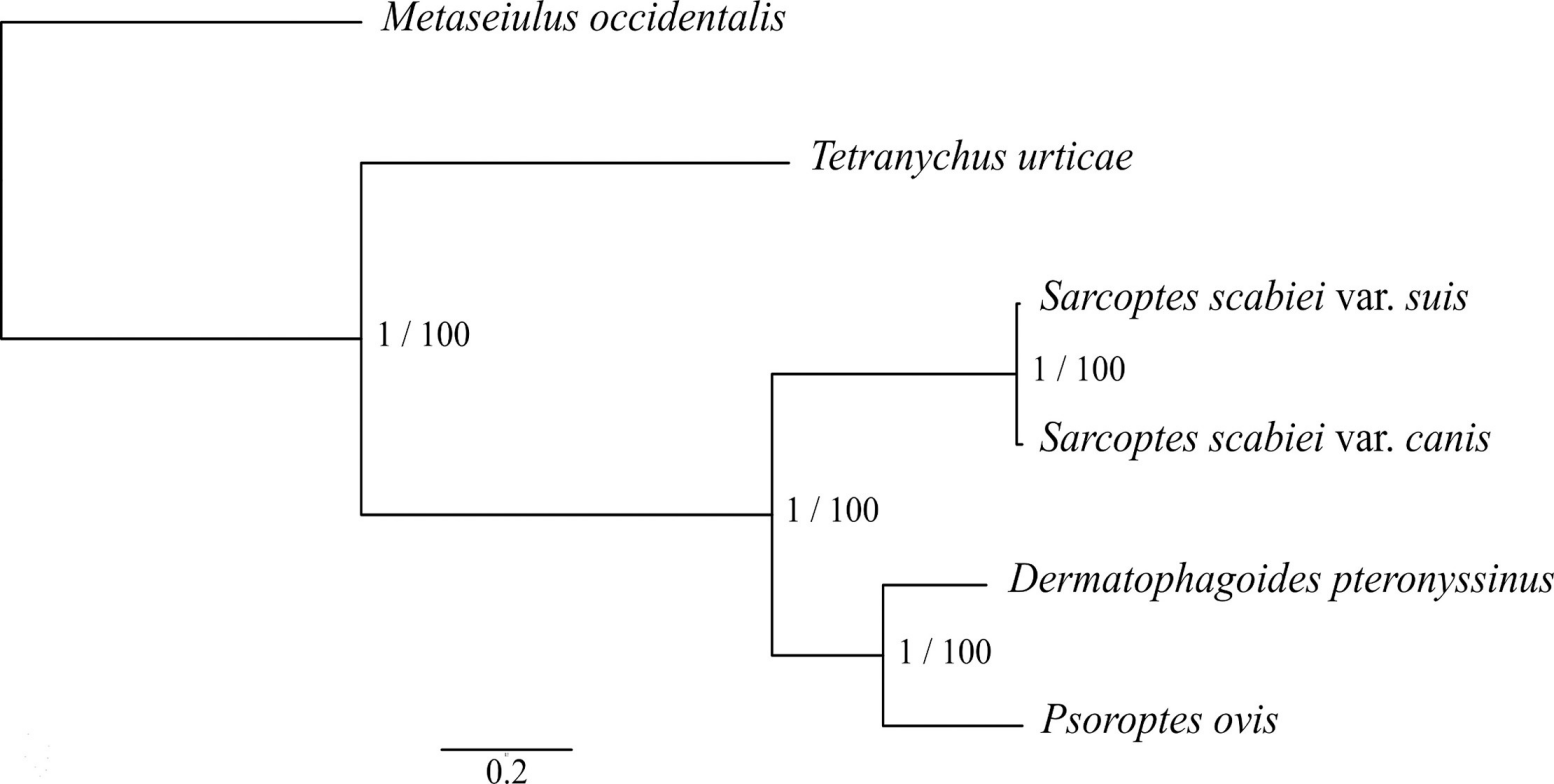
Genetic relationships of selected species of mites. The phylogenetic tree was constructed using data for shared single-copy orthologous protein sequences (n = 2,314) representing *Sarcoptes scabiei* var. *suis*, *Sarcoptes scabiei* var. *canis*, *Dermatophagoides pteronyssinus* (dust mite), *Psoroptes ovis* (sheep mite), *Tetranychus urticae* (spider mite) and *Metaseiulus occidentalis* (predatory mite). All nodes had absolute support values (posterior probability = 1 and bootstrap support = 100%) for both the Bayesian and maximum likelihood inference methods.

We studied the molecular phylogenetic relationships of select free-living and parasitic mite species for which comparative genomic sequence data sets were available. Using data for protein-encoding single-copy orthologous genes (SCOs; n = 2,314), we showed that *S*. *scabiei* var. *suis* is genetically similar to *S*. *scabiei* var. *canis*, phylogenetically related to the dust mite (*Dermatophagoides pteronyssinus*) and the scab mite (*Psoroptes ovis*), and is distant from the spider mite (*Tetranychus urticae*) and the predatory mite (*Metaseiulus occidentalis*) (Fig 3). These relationships are in accord with the numbers of shared orthologous genes, with *S*. *scabiei* var. *suis* sharing most (n = 7,685) with *S*. *scabiei* var. *canis* and least (n = 5,016) with *T*. *urticae* (Fig 2). Density diagrams for coding sequence-, exon- and intron- lengths of *S*. *scabiei* var. *suis* were compared with those of *S*. *scabiei* var. *canis*, *D*. *pteronyssinus* and *T*. *urticae*. The distributions for *S*. *scabiei* were most similar to those for *D*. *pteronyssinus*; the distributions reflected long introns in *T*. *urticae* and short coding regions in *S*. *scabiei* var. *canis* compared with the other mite species studied (Fig 1). Previous results from a phylogenetic analysis of 350 astigmatid mite taxa using concatenated sequence data for five house-keeping genes (8942 nt) [27] suggested that a single common ancestor of the pyroglyphid (dust) mites evolved from a permanent, parasitic life style to become secondarily free-living.

### **Intervention target**s

The excessive and uncontrolled use of a small number of drug classes for the treatment of scabies has led to drug resistances to some of these compounds [28]. Unfortunately, only a small number of scabicides, permethrin and ivermectin in particular, have been available for treatment [14, 29–31]. However, these drugs do not kill eggs and have short half-lives in skin. As a foundation to explore novel intervention targets for *S*. *scabiei*, we identified and manually curated some key groups of proteins inferred to be encoded in this mite, including peptidases, peptidase inhibitors, kinases, G-protein coupled receptors (GPCRs) and ion channels.

Peptidases (n = 217) represented five key classes (aspartic, cysteine, metallo-, serine and threonine), with the metallo- (n = 68; 31.3%) and serine peptidases (n = 74; 34.1%) predominating (S3 Table). Notable were excreted peptidases, such as cathepsins (C01A; n = 3), serine peptidases (S09; n = 2), threonine peptidases (T01A; n = 7) and aminopeptidases (M17; n = 2), which are likely to be involved in cutaneous establishment, protein degradation, immune evasion and/or activation of inflammation, based on knowledge of the biology of *S*. *scabiei* [18]. Identified protease inhibitors (n = 30) included immunosuppressive factors, such as cytotoxic T-lymphocyte antigen-2 alpha (I29; n = 7), alpha-2-macroglobulin (I39; n = 3), subtilisin (I08; n = 7) and aprotinin (I02; n = 2), as well as genes homologous to those encoding serpins (I04; n = 2; SAR_2327s and SAR_4743s), which are known to inhibit activation pathways of the human complement system [32] (S4 Table).

Kinases (n = 251) represented mainly the groups CAMK (n = 53), CMGC (n = 26), tyrosine (TK; n = 21), AGC (n = 19), STE (n = 17), TKL (n = 16) and atypical (n = 8) kinases (S5 Table), which have significant potential as drug targets in parasites due to their role in pivotal cellular processes [33, 34]. GPCRs (n = 106) representing the rhodopsin classes A (n = 73), B1 (n = 9), class B2 (n = 7), class C (n = 8), class F (n = 4) (S6 Table) are intensively studied drug targets [35], and are known to bind molecules critically involved in key biological processes including signalling proteins (e.g., chemokines), neuropeptides (e.g., bombesin, galanin, neuromedin U, neuropeptide Y, neurotensin and tachykinin), lipids (e.g., lysophosphatidylinositol and cannabinoid), hormones (e.g., adrenaline, calcitonin, cholecystokinin, corticotropin-releasing, glucagon, oxytocin, gonadotropins, somatostatin, thyrotropin-releasing and vasopressin), amino acids (gamma-aminobutyric acid and metabotropic glutamate) and/or compounds such as acetylcholine, dopamine, histamine and 5-hydroxytryptamine. Since 2012, > 69 drugs that target GPCRs have been approved by the U.S. Food and Drug Administration (FDA) [36]. Ion channel proteins (n = 126), including voltage-gated ion channels (VGICs; n = 27) and ligand-gated ion channels (LGICs; n = 48), were also identified (S7 Table). Such channels are known targets for endo- and ecto-cidal compounds, including permethrin which targets voltage-gated sodium channels (VGSC) [15, 37], and macrocyclic lactones (e.g., ivermectin and moxidectin) which target glutamate-gated chloride channels (GluCls) [16, 30, 31]. We expect some of these peptidases, peptidase inhibitors, kinases, GPCRs and ion channels to be intervention target candidates that warrant detailed evaluation in *S*. *scabiei* in the future.

### The host-pathogen interplay and immunogens/allergens

Excretory/secretory proteins are central to the host-mite relationship [28, 38]. A proteomic analysis of faecal matter from *S*. *scabiei* var. *suis* revealed totals of 236 excretory proteins (representing the ‘excretome’) (S8 Table) and 373 secretory proteins (‘secretome’) (S9 Table), with 14 proteins being common to both protein sets. The excretome includes 20 proteases, including 7 threonine-, 4 metallo-, 4 cysteine-, 4 serine- and 1 aspartic peptidases (S3 Table; S8 Table), and 5 peptidase inhibitors (including 2 immunosuppressive factors representing cytotoxic T-lymphocyte antigen-2 alpha), 2 subtilisin inhibitors and 1 trypsin inhibitor (aprotinin) (S4 Table; S8 Table). Many of these peptidases and inhibitors are likely involved in the degradation/digestion of skin, tissue barriers and nutrients, and also proposed to play critical roles in the growth, development, moulting and survival of *S*. *scabiei* on the host animal and immunomodulation by this mite [28, 38].

We inferred 85 putative allergens (S10 Table) to be encoded in the genome of *S*. *scabiei* var. *suis*, many of which are homologs of known allergens in *D*. *farinae* (22 of 48; 45.8%; S11 Table) and *D*. *pteronyssinus* (20 of 37; 54.0%; S12 Table) [25, 39]. The inferred excretome contained 28 of these homologs, whereas the secretome contained four. Interestingly, the inferred allergens are amongst the most highly-transcribed genes in the genome, and 22 of them appear to be unique to *S*. *scabiei* (S10 Table).

Apolipoprotein, glutathione S-transferases, cysteine- and serine proteases and serine protease inhibitors have been hypothesised as vaccine candidates against scabies [40]. Here, we identified apolipoproteins Ssag1 and Ssag2 [41], the first of which (SAR_333s) is inferred to be an excreted allergen, but the second (SAR_1661s) is not (S10 Table). We inferred a previously-discovered glutathione S-transferase [42] to be an allergen (SAR_5548); of 11 other glutathione S-transferases identified here, 8 are likely allergens, 3 of which are predicted to be excreted (S8 Table; S10 Table). We also identified a serine protease (cf. accession no. AY333071), an inactive cysteine protease (AY525155) and an active cysteine protease (AY525149) [43, 44], all of which are inferred to be allergens (SAR_9234s, SAR_6923s and SAR_5356s, respectively) (S3 Table). We also identified two serine protein inhibitors (serpins; accession nos. JF317220.1 and JF317222.1) [32], one of which is inferred to be an allergen (SAR_4743s; S4 Table) and the other (SAR_1449s) not.

### Functional genomics and double-stranded RNA interference (RNAi) machinery

Prioritised target candidates (S10 Table) could first be tested for essentiality in *S*. *scabiei* using RNAi, which might support the development of a scabicide. Moreover, functional analysis of the ~ 22% of *S*. *scabiei* protein-encoding genes proposed to be parasite-specific, some of which might be involved in host-parasite interactions, could be facilitated by gene knockdown experiments. The recent establishment of an RNAi assay for *S*. *scabiei* [45] should underpin integrative functional genomic, transcriptomic and proteomic analyses [46] of distinct stages of *S*. *scabiei* in the future. To provide a foundation for such studies, we explored RNAi pathways in this mite.

**Fig 4:**
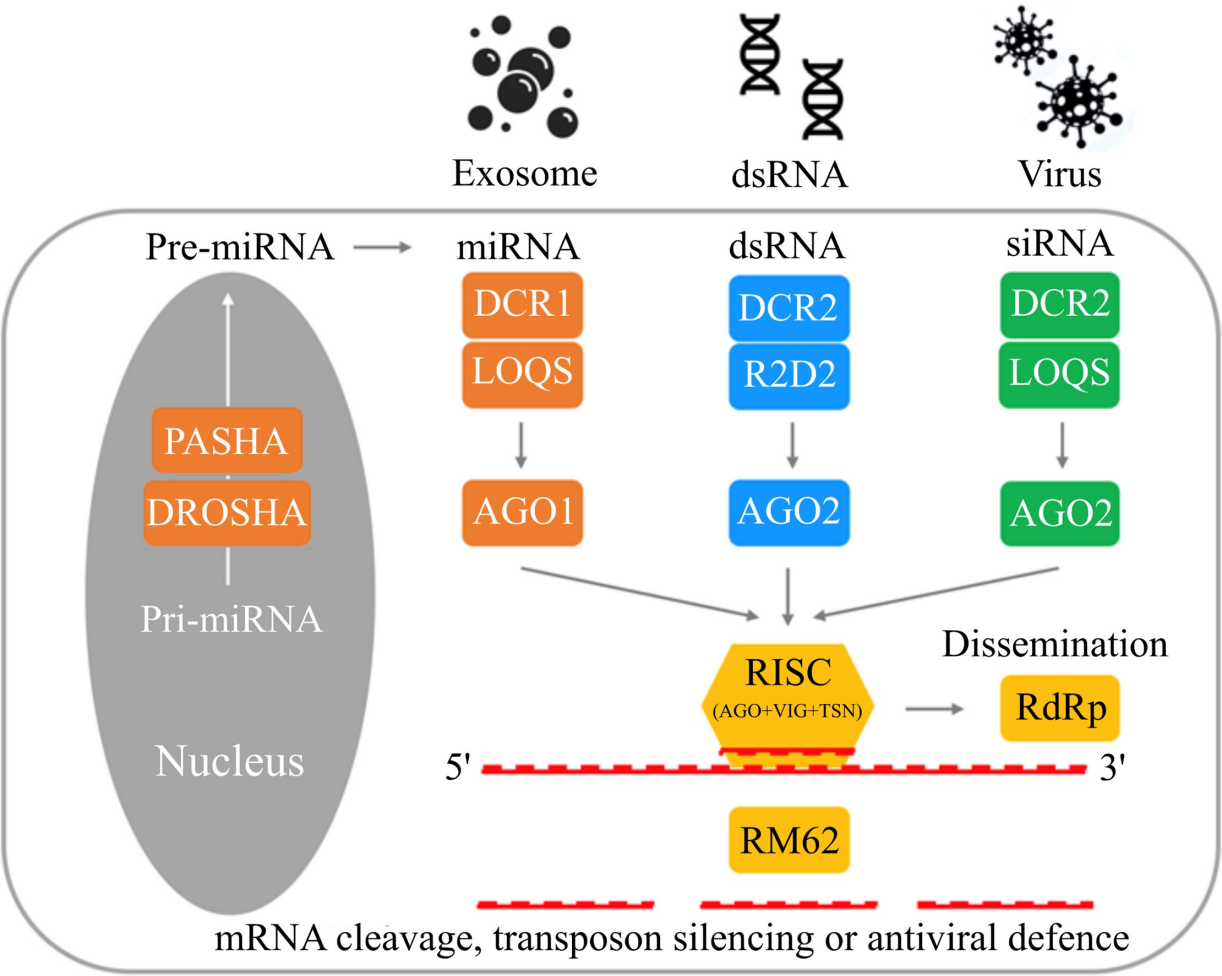
Proposed RNA interference machinery of *Sarcoptes scabiei*. Proteins PASHA and DROSHA are involved in the endogenous synthesis of miRNA. Endogenous or exogenous miRNA, dsRNA and viral siRNA are recognised and diced by endoribonucleases DCR1 or DCR2, mediated by proteins LOQS or R2D2, and transferred to argonaut protein (AGO1 or AGO2), forming the RNA-induced silencing complex (RISC). The RISC facilitates targeting specific transcripts, leading to mRNA cleavage and antiviral defence *via* ATP-dependent RNA helicase (RM62). The silencing effect can be disseminated to other cells *via* a key component RNA-dependent RNA polymerase (RdRp); miRNA, dsRNA and virus-derived siRNA pathways are indicated in orange, blue and green, respectively. Silencing and dissemination modules are indicated in yellow.

Typically, the RNAi machinery of eukaryotic organisms comprises the canonical microRNA (miRNA), small-interfering RNA (siRNA) and/or piwi-interacting RNA (piRNA) pathways [47, 48]. These RNAi pathways regulate a range of biological processes at post-transcriptional level via essential cofactors, the Dicer- and Argonaute-family proteins [49, 50]. Although RNAi pathways have been defined in the model arthropod *Drosophila melanogaster* [51], very little is known about them in *S*. *scabiei*. Here, we identified gene homologues (*n* = 29) encoding core components of RNAi pathways in *S*. *scabiei* (S13 Table). The results revealed relatively conserved miRNA, dsRNA, viRNA and/or piRNA pathways (Fig 4). Although components [i.e., systemic RNAi defective gene (*sid*), synthetic secondary siRNA-deficient argonaut mutant (*sago*) and RNAi spreading defective gene (*rsd*)] that are known to function in dsRNA/siRNA uptake and secondary siRNA dissemination in nematodes [52] were not detected in *S*. *scabiei*, the presence of the RNA-dependent RNA polymerase coding gene (*rdrp*) suggested an endogenous synthetic machinery for secondary siRNAs, which might link to a novel spreading mechanism. In addition, although homologous piRNA-binding proteins aubergine (AUB) and PIWI were not detected (Fig 4), the genes *ago-1*, *-2* and/or *-3* encoding similar protein domains to those of AUB and PIWI may play complementary roles in a piRNA-like pathway in *S*. *scabiei*. The lack of a canonical piRNA pathway in *S*. *scabiei* is consistent with findings for dust mites [53].

### Concluding remarks

The present genomic and molecular exploration of *S*. *scabiei* provides improved insights into the molecular landscape of one of the most important mite pathogens of animals worldwide. This study has inferred molecules involved in host-parasite interactions and immune responses/allergy. The improved genome assembly and associated data sets for *S*. *scabiei* should accelerate post-genomic explorations of molecules involved in mite reproduction and development, metabolism, parasite-host interactions, disease pathogenesis, and the genetics and mechanisms of drug resistance.

Inferring the RNAi machinery in *S*. *scabiei* could assist functional genomic work on selected stages (e.g., eggs) of the parasite. Given that gene-specific knockdown by double-stranded RNA interference (RNAi) has been demonstrated [45], we believe that genome-assisted drug target or drug discovery could provide a complementary approach to the screening of whole mites for new scabicides, similar to approaches proposed for parasitic helminths [54]. The aim is to identify genes or molecules whose inactivation by one or more drugs would selectively kill *S*. *scabiei* but not harm the host animal. Combined with the bioinformatic prediction and prioritisation of essential genes from functional information (e.g., lethality) available for other metazoan organisms, particularly *D*. *melanogaster*, using machine learning approaches [55], RNAi-based screening of *S*. *scabiei* stages provides a powerful functional genomics tool to validate prioritised targets. Focusing on groups of molecules, such as the complex array of peptidases, GPCRs, kinases and ion channels, and understanding their involvement in the host-mite interplay would likely assist in the design of new drugs or a vaccine against scabies. Moreover, future studies should focus on defining a spectrum of key molecules involved in pathways associated with the development of the nervous system in different life-stages of the mite, and on evaluating their potential as drug targets. The availability of a gene knockdown system [45], a drug screening platform [56, 57] and an *in vivo* pig-scabies model [58] provide a particularly useful context to assess prioritised intervention targets and then to evaluate drug candidates both *in vitro* and *in vivo*. Although the present study focused on *S*. *scabiei*, the results and methods employed here should be readily applicable to other ectoparasites of major animal and human health importance. We believe that the substantially improved genome of *S*. *scabiei* should accelerate both fundamental and applied investigations of scabies, enabling the development of new interventions for this important neglected tropical disease.

## Materials and methods

### Ethics approval

Animal ethics approval was granted by the QIMR Berghofer Medical Research Institute (permit nos. P630 and P2159) and the Ethics Committee of the Queensland Animal Science Precinct (permit SA 2015/03/504).

### Production and procurement of *S*. *scabiei*

*Sarcoptes scabiei* was produced on pigs (3 months of age), isolated and stored using a well-established protocol [21]. Mites (n = 1000; approximately equal proportion of larvae, nymphs and adults) were isolated from skin crusts from *S*. *scabiei*-infected pigs, washed extensively, and directly snap frozen and stored at -70°C. In addition, faecal samples (n = 5) were collected from five different batches of mites (same number and stages) isolated from skin crusts taken from pigs on different days; from these faecal samples, crude protein extracts were prepared, freeze-dried and resuspended in 200 μl 8M urea in 100 mM triethylammonium bicarbonate (pH 8.5) with protease inhibitor cocktail set I (Merck, Denmark) [59].

### Genomic DNA library construction and sequencing

High molecular weight genomic DNA was isolated from six samples each containing 1,000 motile adults, nymphs, larvae and eggs, collected on different days, using the Gentra Puregene Tissue Kit (Qiagen) according to manufacturer’s instructions. Total DNA amount was determined using a *Qubit* fluorometer dsDNA *HS Kit (Invitrogen)*, *according to the manufacturer’s instructions*. *Genomic* DNA integrity was verified by agarose gel electrophoresis and using a Bioanalyzer 2100 (Agilent). Long-read sequencing of libraries constructed using the 20 kb Template Preparation employing BluePippin Size-Selection System was conducted using an established Pacific Biosciences (PacBio) protocol [60]. Short-read paired-end (PE) libraries (100 bp-inserts) were constructed, checked for size distribution and quality using Bioanalyzer 2100 and sequenced with Illumina HiSeq 2500 using an established method [20]. Jumping libraries (with 3-, 5-, and 7-kb inserts; see S1 Table) were constructed and sequenced using an established method [61]. Library preparation and long-read sequencing was conducted at the Centre for Clinical Genomics at the Translational Research Institute, Diamantina Institute in Wooloongabba, Queensland, Australia. Library preparation and long-read sequencing was conducted using a 20Kb PacBio RSII, Bluepipin size-selected SMRT bell library preparation and sequencing on 10 SMRT cells. The average number of reads per SMRT cell was 51,128 bp; the mean read length was 12,663 bp, and the N50 read length was 18,857 bp.

### RNA isolation and RNA-seq

Total RNA was isolated separately from eggs (n = 16,000) and mixed larvae, nymphs and adults (n = 16,000) of *S*. *scabiei* var. *suis* employing the ToTally RNA Kit (Ambion). RNA yields were estimated spectrophotometrically (NanoDrop 1000), and the integrity of RNA was verified using a BioAnalyzer 2100 (Agilent). Following mRNA isolation using the MicroPolyAPurist kit (Ambion), RNA-seq was carried out as described previously [20]. Sequence data were assessed for quality and adaptors removed.

### Liquid chromatography/tandem mass spectrometry (LC-MS/MS) analysis

The proteome of faecal matter (“excretome”) from *S*. *scabiei* eggs, nymphs and adults was investigated using an established in-solution digestion protocol [62]. In brief, the five samples (i.e. biological replicates; 50 μg of protein each) were reduced, alkylated and double-digested with Lys-C/trypsin mix (Promega, USA) at 37°C for 16 h. The tryptic samples were then acidified with 1.0% (v/v) formic acid and purified using Oasis HLB cartridges (Waters, USA). Using an established technique [63], tryptic peptides were analysed using a Q Exactive Plus Orbitrap mass spectrometer (Thermo Fisher, USA). Protein- and peptide- level fractionation and LC-MS/MS analysis of whole mite preparations was undertaken at the Institute of Bioinformatcs ain Bangalore, India, and egg preparations underwent on-tip strong-cation exchange chromatography-based fractionation and were analyzed on Orbitrap Fusion Lumos mass spectrometer interfaced with Easy nLC 1200 UPLC system (Thermo Scientific, Bremen, Germany) at Johns Hopkins University.

### Excretory/Secretory proteins and allergens

Excretory/secretory proteins were inferred from LC-MS/MS (faecal matter) data against the proteome inferred from the genome of *S*. *scabiei*. First, raw LC-MS/MS data were processed with the program MaxQuant using the Andromeda search engine [64]. Fixed modifications of carbamidomethylation of cysteine (+57 Da) and variable modifications of methionine oxidation (+16 Da) were used. Results were compiled at targeted false discovery rate (FDR) of < 0.01 on both the peptide spectrum match (PSM) and the protein level. Proteins identified with ≥ 2 peptides were accepted. Secreted proteins were predicted using the programs SignalP 4.0 [65] and MultiLoc2 [66]. To classify a secreted protein, a predicted signal peptide and predicted extracellular location were required. Allergens were identified using BLASTp v2.2.30+ searches (E-value ≤ 10^−8^) against the NCBI protein nr database, the allergens identified for *S*. *scabiei* var. *canis* [19], and known allergens of *Dermatophagoides farinae* and *D*. *pteronyssinus* [67]; gene models of identified allergens were manually curated using available transcriptomic data.

### Genomic assembly

An established pipeline [68] was used to create an assembly from PacBio sequence read data. In brief, these data were assembled using the program Canu v1.6 [69], polished using both PacBio raw reads and Illumina PE reads employing the programs SmrtLink v5.0.1 [70] and Pilon v1.22 [71], and sequences representing redundant haplotypes were removed using the program HaploMerger2 (build_20160512) [72]. The assembly was then scaffolded using Illumina mate-pair reads (3-, 5- and 7-kb inserts), and gaps were closed with Illumina PE reads in two iterations employing the programs SSPACE v3.0 [73] and GapCloser v1.12 [74].

### Gene prediction

The *S*. *scabiei* protein-coding gene set was inferred utilizing available evidence data, including the transcriptomic data for egg and mixed-sex, motile stages, and protein sequence data were deposited in the UniProtKB/SwissProt database (May 14, 2019) [23]. First, known interspersed repeats in Repbase v.17.02 [75] and simple repeats were masked using the program RepeatMasker [76]. Transcriptomic evidence data were collected from both cDNA [77, 78] and RNAseq experiments; cDNA sequences were assembled using the program CAP3 (version 10/15/07) [79] and RNAseq data using the program Trinity v2.4.0 [80]. CAP3-assembled transcripts were concatenated with *de novo* and genome-guided transcript assemblies acquired using the Trinity pipeline. Transcripts with unknown nucleotide positions (“Ns”) were removed, and cd-hit-est [81] was used to reduce transcript redundancy by 1%. Open reading frames (ORFs) were inferred from the remaining 99% of transcripts employing the program TransDecoder [80], and cd-hit-est was used to reduce redundancy by 1%. This final set of ORFs (≥ 500 bp in length) was used as transcriptomic evidence data for gene predictions and mapped to the genome using BLAT [82]. The validity of splice sites was verified, and ORF-sequences were then used to train the *de novo*-gene prediction program AUGUSTUS [83] that produces a Hidden Markov Model (HMM) for gene prediction. The non-redundant ORFs and the proteome of *T*. *urticae* were also given to MAKER3 [84] to provide evidence for predicted genes. The resultant HMM, the ORFs and the proteome were subjected to analysis using MAKER3 to provide a consensus set of genes for *S*. *scabiei*. Genes inferred to encode peptides of ≥ 30 amino acids in length were preserved. Next, the PASA pipeline [85] employed non-redundant ORFs to improve predicted gene models in three iterations. The gene set was compared against original MAKER3 gene models, and those that did not overlap with the PASA-improved gene models were added to the gene set. Isoforms were removed from this gene set by preserving the longest isoform to represent each gene. For NCBI submission, UTR-regions were removed, and the gene set was verified using the programs GAG v2.0.1 [86] and tbl2asn [87].

### Functional annotation

First, following the prediction of the protein-coding gene set for *S*. *scabiei*, each inferred amino acid sequence was assessed for conserved protein domains using InterPro (release 75.0) [88] employing default settings. Then, amino acid sequences were subjected to BLASTp (E-value ≤ 10^−8^) against the following protein databases: Swiss-Prot within UniProtKB [23]; Kyoto Encyclopedia of Genes and Genomes (KEGG) [89, 90]; and NCBI protein nr [91]. Genes encoding proteases, protease inhibitors, G-protein-coupled receptors (GPCR), kinases and ion channels were manually curated.

### Curation of gene annotations for key protein groups

Gene models were curated employing protein domain architecture information from the InterPro database (release 75.0) and from transcriptomic data. Kinase gene models were curated using an established approach [92]–i.e. kinases were first inferred and classified into groups, families and subfamilies using Kinannote [93], and the PANTHER [94] and InterPro databases were then employed for unclassified kinases. GPCR gene models were identified and manually curated using an established approach [95] and assigned to class, family and/or subfamily based on information from GPCRdb (March 2019 release) [96]. Peptidase gene models were inferred by searching MEROPS peptidase and peptidase inhibitor databases (release 12.1) (BLASTp; E-value ≤ 10^−8^) [97] and manually curated. Ion channel gene models were manually curated and classified based on information from the PANTHER (release 14.1), Pfam (release 32.0) [98] and InterPro (release 76.0) databases.

### Prediction of repeat regions

Genomic repeats specific to *S*. *scabiei* were inferred using the program RepeatModeler [99] that merges repeat predictions from the programs RECON [100] and RepeatScout [101]. Custom repeats and known repeats in Repbase v.17.02 [75] were then masked in the *S*. *scabiei* genome assembly using the program RepeatMasker [76].

### Inferred protein sequence homology

Homologs among *S*. *scabiei*, *T*. *urticae* and *D*. *pteronyssinus* were inferred by comparison among all proteins using the program OrthoMCL v2.0.4 (BLASTp; E-value ≤ 10^−8^). The counts for shared homologous genes among these species were displayed in a Venn diagram.

### Phylogenetic analysis

Single-copy orthologous (SCO) genes were inferred from homologous genes shared by *S*. *scabiei* var. *suis*, *S*. *scabiei* var. *canis* [19], *D*. *pteronyssinus* [25], *Metaseiulus occidentalis* [102], *Psoroptes ovis* [103] and *Tetranychus urticae* [26], and conceptually translated into amino acid sequences. The 1,859 clusters of SCO sequences representing all six species were individually aligned using the program AQUA [104], employing the programs MUSCLE v3.8.31 [105] and MAFFT v.7.271 [106] for the alignment and RASCAL v1.34 [107] for the refinement of alignments. Each gene cluster of SCO sequences with an alignment score of ≥ 0.8 obtained from the program NorMD [108] were merged using the program PartitionFinder v2.1.1 [109] to assign each merged partition to a replacement matrix. Partitions that did not contain all 20 amino acids, or represented mitochondrial or viral sequences, were removed. Remaining partitions were then subjected to separate phylogenetic analyses using the Bayesian inference (BI) and maximum likelihood (ML) tree-building methods. BI analysis was conducted using the program MrBayes v3.2.6 [110] from four independent Markov chains, run for 1,000,000 metropolis-coupled MCMC iterations, for which trees were sampled every 1000 iterations. The resultant tree was inferred by, first, discarding 250,000 sampled trees (25%) as burn-in, and using the remaining sampled trees to infer tree topology, branch lengths and to calculate Bayesian posterior probabilities (BPP). ML analysis was conducted using the program RAxML v8.2.6 [111] and the same replacement matrices were used as for BI analysis. The phylogram was prepared using FigTree v.1.31 (http://tree.bio.ed.ac.uk/software/figtree).

### Density diagrams of gene features

Density diagrams were created using standard commands in the R language [112]. Gene-, exon- and intron- lengths were inferred from the gene models of *D*. *pteronyssinus* [25], *S*. *scabiei* var. *canis* [19], *S*. *scabiei* var. *suis* and *T*. *urticae* [26].

## Supporting information

S1 Table Read and repeat data statistics for *Sarcoptes scabiei*. (XLSX)

S2 Table Annotation for all predicted genes. (XLSX)

S3 Table Predicted proteases for *Sarcoptes scabiei*. (XLSX)

S4 Table Predicted kinases for *Sarcoptes scabiei*. (XLSX)

S5 Table Predicted G protein-coupled receptors for *Sarcoptes scabiei*. (XLSX)

S6 Table Predicted Ion channels for *Sarcoptes scabiei*. (XLSX)

S7 Table Excreted proteins of *Sarcoptes scabiei*. (XLSX)

S8 Table Putative secretome of *Sarcoptes scabiei*. (XLSX)

S9 Table Predicted protease inhibitors for *Sarcoptes scabiei*. (XLSX)

S10 Table Known and putative allergens of *Sarcoptes scabiei*. (XLSX)

S11 Table Sarcoptes scabiei homologs to WHO/IUIS allergens of *Dermatophagoides farinae*. (XLSX)

S12 Table Sarcoptes scabiei homologs to WHO/IUIS allergens of *Dermatophagoides pteronyssinus*. (XLSX)

S13 Table RNA interference pathway components in *Sarcoptes scabiei*. (XLSX)
